# Are you Coupled? Hemodynamic Phenotyping in Pulmonary Hypertension

**DOI:** 10.1093/function/zqac036

**Published:** 2022-07-07

**Authors:** Samuel H Friedman, Ryan J Tedford

**Affiliations:** Division of Pulmonary, Critical Care, Allergy and Sleep Medicine, Medical University of South Carolina, Charleston, SC 29425, USA; Division of Cardiology, Department of Medicine, Medical University of South Carolina, Charleston, SC 29425, USA

*A Perspective on “Multimodality Deep Phenotyping Methods to Assess Mechanisms of Poor Right Ventricular–Pulmonary Artery Coupling*.”

*“It's not the load that breaks you down, it's the way you carry it”–Lou Holtz*

For decades our approach in medicine has been to classify diseases based on diagnostic algorithms, with the aim of applying specific therapies to large groups of patients. As our understanding of pathophysiology has evolved, we have come to realize that diseases vary on the patient level. The one-size-fits-all treatment methodology is limiting, and an individualized patient-centered management plan is the ultimate goal.

Pathophysiologic diversity is especially evident in disorders of the pulmonary circulation and the right ventricle (RV). Pulmonary hypertension (PH) is currently divided into World Health Organization (WHO) groups based on the perceived etiology of the vascular insult: pulmonary arterial hypertension (PAH, WHO Group 1), PH due to left heart disease (WHO Group 2), PH due to chronic lung disease (WHO Group 3), PH due to pulmonary artery obstructions (WHO Group 4), and PH due to unexplained or multifactorial mechanisms (WHO Group 5).^1^ Accordingly, therapeutic interventions to address PH differ significantly between groups. However, this classification is less than perfect as there is not only considerable heterogeneity within each group but also overlap between groups. A large multicenter effort to better phenotype PH is currently underway to address these limitations.^2^

Central to the diagnosis and classification of PH (and ultimately treatment of PH) is hemodynamic assessment by right heart catheterization. This standard assessment measures biventricular filling pressures, pulmonary pressures and cardiac output. In recent years, more advanced hemodynamic techniques have been developed. Coupled with advanced cardiac imaging, these modalities represent an opportunity to both (1) improve our understanding of pathophysiology and (2) more deeply phenotype PH patients with the goal of developing precise therapeutic strategies.

In this issue of *Function*, Raza et al. leverage their expertise in advanced hemodynamics to more robustly assess the relationship between the RV and pulmonary circulation.^3^ The authors show the heterogenous physiology uncovered in four distinct disease states: PAH, chronic thromboembolic pulmonary hypertension, PH due to heart failure with preserved ejection fraction (PH-HFpEF), and noncardiogenic dyspnea. Each patient underwent invasive cardiopulmonary exercise testing, pulmonary impedance measurement (the most comprehensive description of RV afterload), echocardiography with strain imaging, cardiac magnetic resonance imaging, and right ventricle pressure–volume (PV) analysis at both rest and during exercise. PV relations are the gold standard to assess ventricular function: the coupling of load independent RV contractility to the afterload imposed on it from the pulmonary circulation (so-called, RV–PA coupling) as well as diastolic function.^4^ The distinct differences appreciated in the four patients highlight the possibility of individualized phenotyping in this complex disorder.

PH-HFpEF is a prime example of a condition that may benefit from this type of comprehensive hemodynamic evaluation. It is currently subcategorized by resting hemodynamic assessment into isolated postcapillary PH (IpcPH) and combined post- and precapillary PH (CpcPH). While both require elevated left atrial pressures, CpcPH is distinguished from IpcPH by the presence of elevated pulmonary vascular resistance, and is associated with more RV dysfunction and worse clinical outcomes.^5^ Despite sharing some hemodynamic similarities with PAH, trials of PAH therapies in this population have largely been unsuccessful.^6^ Patient selection may in part explain the lack of benefit. For example, those patients with a combination of high pulmonary impedance and an “at-risk” RV (RV–PA uncoupling at rest or with exercise), which leads to relative LV underfilling during exercise, may be a group more likely to benefit from RV afterload reduction therapies. On the other hand, patients with preserved RV–PA coupling but predominate left ventricular dysfunction during provocation (the HFpEF-PH patient in this study) could benefit more from left atrial decongestive strategies. These patients are not always readily distinguishable with standard resting hemodynamics and imaging.

Advanced hemodynamics may also prove useful in risk stratification. At first glance, the PAH patient in this study may appear well compensated by traditional evaluation with a normal resting RV ejection fraction and reasonable cardiac output. However, PV analysis shows evidence of significant RV–PA uncoupling at rest and evaluation during exercise confirms poor cardiac reserve along with marked functional limitation. This phenotype has been associated with worse outcomes (coupling ratio <0.65–0.70), and escalation of PAH therapy in this case may be warranted.^7–9^

Although we sincerely congratulate the authors for the exciting application of these hemodynamics and imaging techniques, we believe several points merit further discussion. This case series allows us to appreciate what *can* be done, but we are of course unable to draw specific conclusions about the broader populations represented by these four patients. Techniques like PV analysis and measures of impedance are likely not practical for standard clinical practice given the expense of equipment and expertise to both acquire and interpret the results. Less invasive surrogates for both measures have been developed and are actually utilized in this study: single beat estimations of PV relations and impedance estimated from echo-based flow measures. Although both have shown associations with the gold standard measures, the use of these surrogates may be most appropriate for larger outcome studies rather than to compare and contrast physiologic differences between small groups of patients or especially in individual patients where precision is critical.^9,10^ Given the potential subjectivity in interpretation of these data, more validation is likely required before they can be used for such a purpose.

In summary, the study by Raza and colleagues provides a beautiful demonstration of what may be potentially learned from a comprehensive physiologic assessment utilizing multiple hemodynamic and imaging modalities. In particular, evaluation during exercise provocation may be additive to diagnostic, prognostic, and therapeutic strategies in PH. With future work like this, we may be able to tailor therapies to not only reduce the “load that breaks you down” but also improve the way the RV “carries it.”

## Funding

None declared.

## Data availability

There are no data presented in this editorial/perspective
